# Patterns of adolescent physical activity and dietary behaviours

**DOI:** 10.1186/1479-5868-6-45

**Published:** 2009-07-22

**Authors:** Natalie Pearson, Andrew J Atkin, Stuart JH Biddle, Trish Gorely, Charlotte Edwardson

**Affiliations:** 1 School of Sport, Exercise & Health Sciences, Loughborough University, Loughborough, Leicestershire, UK

## Abstract

**Background:**

The potential synergistic effects of multiple dietary and physical activity behaviours on the risk of chronic conditions and health outcomes is a key issue for public health. This study examined the prevalence and clustering patterns of multiple health behaviours among a sample of adolescents in the UK.

**Methods:**

Cross-sectional survey of 176 adolescents aged 12–16 years (49% boys). Adolescents wore accelerometers for seven days and completed a questionnaire assessing fruit, vegetable, and breakfast consumption. The prevalence of adolescents meeting the physical activity (≥ 60 minutes moderate-to-vigorous physical activity/day), fruit and vegetable (≥ 5 portions of FV per day) and breakfast recommendations (eating breakfast on ≥ 5 days per week), and clustering patterns of these health behaviours are described.

**Results:**

Boys were more active than girls (p < 0.001) and younger adolescents were more active than older adolescents (p < 0.01). Boys ate breakfast on more days per week than girls (p < 0.01) and older adolescents ate more fruit and vegetables than younger adolescents (p < 0.01). Almost 54% of adolescents had multiple risk behaviours and only 6% achieved all three of the recommendations. Girls had significantly more risk factors than boys (p < 0.01). For adolescents with two risk behaviours, the most prevalent cluster was formed by not meeting the physical activity and fruit and vegetable recommendations.

**Conclusion:**

Many adolescents fail to meet multiple diet and physical activity recommendations, highlighting that physical activity and dietary behaviours do not occur in isolation. Future research should investigate how best to achieve multiple health behaviour change in adolescent boys and girls.

## Introduction

Poor diet and physical inactivity are established risk factors for chronic disease. In young people, physical activity and healthy diets including regular breakfast consumption and adequate levels of fruit and vegetables, have important short- and long-term health protective effects. For example, physical activity in young people may benefit cardiovascular disease (CVD) risk factors, adiposity and bone health, which could influence health in adulthood [1]. Child fruit and vegetable consumption has been associated with a lower incidence of respiratory symptoms [2] and appears to be protective against cancer in adulthood [3]. Also, cross-sectional and longitudinal research has shown that young people who regularly eat breakfast are less likely to be overweight than those who skip breakfast [4,5]. Despite such health benefits, young people are more likely to skip breakfast than any other meal [6] and data from the Health Behaviour in School-aged Children (HBSC) study shows that less than two-fifths of young people eat fruit daily, and only about a third eat vegetables daily [7]. Also, recent data suggest that 30% of boys and 40% of girls in the United Kingdom (UK) do not meet the current physical activity guidelines [8]. Health risks associated with individual behaviours are broadly acknowledged, yet, arguably a key issue for public health is that there is potential for synergistic effects of multiple health behaviours on the risk of chronic conditions and health outcomes [9,10]. However, little is known about the relationship among these behaviours and their clustering patterns among adolescents in the UK.

There is evidence of an association between physical activity and dietary behaviours in adolescents. For example, Kremers et al. [11] and Driskell et al. [12] found an association between low fruit and vegetable consumption and low levels of physical activity. Keski-Rahkonen et al. [13] and Cohen et al. [14] found that breakfast skipping was associated with infrequent physical activity. Evidence for the clustering of two or more health behaviours in adolescents comes mainly from the US. Pronk et al. [15] found that only 31% of adolescents met the recommendations for adolescent-specific healthy lifestyle factor guidelines (including healthy weight, no smoking, physical activity and high quality diet). Sanchez et al. [16] found that nearly 80% of 11–15-year olds had multiple risk factors related to diet (fruit and vegetable consumption and calories from fat), physical activity and sedentary behaviour (TV time and moderate-to-vigorous-physical activity – MVPA). The tendency for physical activity and dietary behaviours to cluster has important implications for health promotion, highlighting the need for effective behaviour change interventions targeting multiple behaviours [12].

Targeting change in multiple behaviours offers the potential of increased health benefits, maximized health promotion, and reduced health care costs. Success in changing one or more lifestyle behaviour may also increase confidence or self-efficacy to improve risk behaviours that individuals have low motivation to change, and as such, health behaviour change may serve as a gateway to overall healthful lifestyle change [17]. Given that adolescence is a critical period for the adoption of health behaviours, and that lifestyle habits and attitudes adopted during this life phase may track into adulthood [18], an improved understanding of health behaviour clustering among adolescents could identify high-risk groups and inform strategies for multiple health behaviour interventions.

Very few studies examining clustering of health behaviours among adolescents have used objective measures of physical activity [16], with studies usually relying on self-report methods. To our knowledge there is currently no published research examining whether breakfast consumption clusters with fruit and vegetable consumption and physical activity in a sample of adolescents from the UK. The current study aims to develop and add to the current literature by examining the prevalence and clustering patterns of multiple health behaviours among a sample of adolescents from the UK. This study will focus specifically on objectively assessed physical activity, plus fruit and vegetable consumption and breakfast consumption.

## Methods

### Sample and procedure

Cross-sectional data were collected between October 2007 and June 2008. Study procedures were approved by the Ethical Advisory Committee of the host university. Data were obtained from adolescents (12–16 years) recruited from three secondary schools in the East Midlands region of the UK. Staff at participating schools selected a subset of their classes for participation. All students from nominated classes (n = 363) were eligible and received written information on the project. Consent was sought from parents prior to the study and adolescent participants provided assent before completing written surveys during class. Participants completed questionnaires during Physical Education or Personal, Social and Health Education (PHSE) lessons, under the supervision of trained researchers and class teachers.

### Measures

Demographic information, including date of birth and ethnicity, was provided by the school. Questionnaires collected information on demographic characteristics of adolescents including gender and home postcodes. Socio-economic status (SES) was determined using the Index of Multiple Deprivation (IMD), a measure of compound social and material deprivation, calculated from a variety of data including income, employment, health, education, and housing. It is based on the postcode of the participant's home, and thus represents an area level approximation of SES.

#### Adolescent dietary behaviour

Adolescent food intake was assessed using a 30-item food frequency questionnaire (FFQ), based on the previously validated Youth/Adolescent Food Frequency questionnaire (YAQ) [19]. Adolescents were asked how often they ate ten different fruits and twelve different vegetables in the past month. Responses to questions on the frequency of consumption of specific fruits and vegetables were summed to compute total frequency of fruit and vegetable consumption/day, respectively. For the purpose of this study each item was summed to calculate the frequency of consumption of 'fruits and vegetables' (FV) per day. Guided by the current recommendations for fruit and vegetable consumption (5 portions of FV/day) [20], the total daily frequency of FV consumption was dichotomised into <5 times/day or ≥ 5 times/day.

Breakfast consumption was assessed with a single-item asking adolescents how often they ate breakfast in the past seven days. While there are no current national recommendations for frequency of breakfast consumption, evidence suggests that young people who eat breakfast on most days of the week see health benefits (e.g. lower Body Mass Index) compared to those who skip breakfast [4,21]. A recent government initiative in the UK recommends that parents encourage their children to eat breakfast regularly [22]. Given such evidence, frequency of breakfast consumption/week was dichotomised into <5 days/week or ≥ 5 days/week.

#### Physical activity

Physical activity was assessed by Actigraph GT1M accelerometers (ActiGraph, Fort Walton Beach, FL) using a 5 second measurement interval (epoch). Participants were instructed to wear the accelerometer over their right hip for one-week. Exceptions included time spent sleeping, showering and during water-based activities. Duration of MVPA was computed only for adolescents who wore the accelerometer for a minimum of three days [23], defined as days on which accelerometer counts were between 10,000 and 20 million [24].

To estimate the time spent per day in moderate intensity physical activity (3.0–5.9 metabolic equivalent of rest [METs]) and vigorous intensity physical activity (6.0+ METs), age specific movement count thresholds [25] were applied. Time-per-day in MVPA was derived by summing these values across valid days. The proportion of adolescents meeting the physical activity recommendations for young people [26] was calculated according to whether they performed an average of ≥ 60 mins/day of MVPA. Time spent in MVPA was dichotomised as <60 mins/day or ≥ 60 mins/day.

### Statistical analyses

All analyses were conducted using SPSS statistical software version 16.0. Descriptive statistics were used to summarise sociodemographic, physical activity and dietary characteristics of the sample. Adolescents were categorised as younger or older adolescents by dichotomising at the mean (14.4 years). Mann-Whitney tests were performed to examine gender and age-group differences in mean minutes/day spent in MVPA, fruit and vegetable consumption per day, and breakfast consumption per week. The proportion of adolescents achieving ≥ 60 mins/day of MVPA, and the proportion of adolescents consuming fruit and vegetables ≥ 5 times/day, and breakfast ≥ 5 times/week were compared by gender and age-group using Pearson's chi-square (χ^2^) tests of significance.

A total risk behaviour score was calculated for each participant based on the total number of unmet health recommendations (range from zero to three). Pearson's chi-square tests were used to examine gender and age-group differences in the number of risk behaviours. The proportion of adolescents in each multiple risk behaviour combination was determined to examine behaviour clustering patterns, and Pearson's chi-square tests were used to examine gender and age-group differences in the clustering patterns.

## Results

### Sample characteristics

In total, 328 pupils provided consent and completed the questionnaire (90% response rate). Of these, 176 (54%) provided usable accelerometer data. Comparison of these 176 adolescents with those who did not provide usable accelerometer data (n = 152) showed no significant differences in age, SES, ethnicity or fruit and vegetable consumption. However, a significantly (p < 0.01) higher proportion of those with usable accelerometer data were girls, compared to boys (63% compared to 47%), and ate breakfast on more days per week (5.3 days compared to 4.5 days). Thus, our figures likely underestimate the prevalence of not meeting the breakfast recommendations. The final sample composition was 176 adolescents, with 87 boys, 89 girls, 98 younger adolescents and 78 older adolescents. The mean age of younger adolescents was 13.3 years and of older adolescents was 15.6 years. Ninety-five percent of participants were of White ethnic background and 71% were of high SES.

### Adolescent physical activity, fruit and vegetable and breakfast behaviours

There were significant gender differences in MVPA and breakfast consumption (Table 1). Boys engaged in more MVPA per day and ate breakfast on more days per week compared to girls (p < 0.01). Differences were also evident by age-group. Younger adolescents engaged in more MVPA per day compared to older adolescents (p < 0.001). Older adolescents ate more fruit and vegetables per day compared to younger adolescents (p < 0.001).

**Table 1:** Distribution fruit and vegetables consumption per day, breakfast consumption per week and minutes per day spent in moderate-to-vigorous intensity physical activity (MVPA).

	***Total (n = 176)***	***Boys (n = 87)***	***Girls (n = 89)***	***Younger adolescents*** ***(n = 98)***	***Older adolescents*** ***(n = 78)***
**MVPA minutes/day**					
25th percentile	30.5	37.6	25.5**	39.2	22.6***
50th percentile	44.1	49.3	40.5	48.1	38.1
75th percentile	56.4	64.3	53.1	65.1	49.5
					
**Frequency of FV consumption/day**					
25th percentile	3.1	2.8	3.3	2.5	3.8***
50th percentile	4.5	4.8	4.4	3.9	5.4
75th percentile	6.7	6.7	6.9	6.1	7.8
					
**Frequency of breakfast consumption, days/week**					
25th percentile	4	5	2.5**	4	4
50th percentile	7	7	6	7	7
75th percentile	7	7	7	7	7

SD, standard deviation; FV a composite measure of fruit and vegetables.

**P < 0.01; ***P < 0.001. Mann-Whitney tests.

### Prevalence of meeting health recommendations

The proportion of boys, girls, younger and older adolescents meeting the physical activity, fruit and vegetable, and breakfast consumption recommendations are displayed in Table 2. A higher proportion of boys, compared to girls, ate breakfast on more than 5 days/week (p < 0.01). A higher proportion of younger adolescents, compared to older adolescents, met the physical activity recommendations and did not meet the recommendations for fruit and vegetable consumption (p < 0.01). Only 6% of the adolescent sample met the recommendations for all three of the health behaviours and almost 10% had three health risk behaviours. A higher proportion of girls, compared to boys, had three health risk behaviours (p < 0.01).

**Table 2:** Gender and age distribution of meeting health recommendations

	***Total (n = 176)***	***Boys (n = 87)***	***Girls (n = 89)***	***Younger adolescents (n = 98)***	***Older adolescents (n = 78)***
**Meet >60 minutes MVPA per day, n (%)**					
No	137 (78.8)	63 (72.4)	74 (83.1)	68 (69.4)	69 (88.5)
Yes	39 (22.2)	24 (27.6)	15 (16.8)	30 (30.6)	9 (11.5)**
**Meet >5 portions fruits/vegetables, n (%)**					
No	98 (55.2)	45 (51.2)	53 (59.6)	63 (64.3)	35 (44.9)**
Yes	78 (44.8)	42 (48.3)	36 (40.4)	35 (35.7)	43 (55.1)
**Meet >5 days a week eating breakfast, n (%)**					
No	41 (23.3)	13 (14.9)	28 (31.5)	23 (23.5)	18 (23.1)
Yes	135 (76.7)	74 (85.1)	61 (68.5)**	75 (76.5)	60 (76.9)
**Number of risk behaviours, n (%)**					
0	11 (6.2)	9 (10.3)	2 (2.2)	7 (7.1)	4 (5.1)
1	71 (40.3)	40 (46.0)	31 (34.8)	36 (36.7)	35 (44.9)
2	77 (43.8)	33 (37.9)	44 (49.4)	47 (48.0)	30 (38.5)
3	17 (9.7)	5 (5.7)	12 13.5)**	8 (8.2)	9 (11.5)

**P < 0.01. Pearson's chi-square test of significance.

The clustering patterns of the three health risk behaviours are described in Table 3. For adolescents with two risk behaviours, the most prevalent cluster was formed by not meeting the physical activity and fruit and vegetables recommendations. A higher proportion of girls, compared to boys, had the cluster pattern of not meeting the recommendations for physical activity and breakfast consumption (p < 0.01). A higher proportion of boys, compared to girls, and older adolescents, compared to younger adolescents, had the most prevalent single risk factor of not meeting the recommendations for physical activity (p < 0.01). A higher proportion of younger adolescents, compared to older adolescents, had the single risk factor of not meeting the recommendations for fruit and vegetable consumption (p < 0.01).

**Table 3:** Descriptive cluster pattern of multiple risk behaviours

***Number of risk behaviours***	***Percent of sample***	***Boys (n = 87)***	***Girls (n = 89)***	***Younger adolescents (n = 98)***	***Older adolescents (n = 78)***
**3**: All three risk behaviours	9.7	5.7	13.5**	8.2	11.5
**2**: MVPA < 60 minutes/day **and **< 5 fruit/vegetables per day	33.0	31.0	34.8	36.7	28.2
**2**: MVPA < 60 minutes/day **and **< 5 days a week eating breakfast	6.2	1.1	11.2**	5.1	7.7
**2**: < 5 fruit/vegetables per day **and **< 5 days a week eating breakfast	5.7	5.7	5.6	8.2	2.6
**1**: MVPA < 60 minutes/day	29.0	34.5	23.6**	19.4	41.0**
**1**: < 5 fruit/vegetables per day	7.4	9.2	5.6	11.2	2.6**
**1**: < 5 days a week eating breakfast	2.8	2.3	3.4	4.1	1.3
**0**: No risk behaviours	6.2	10.3	2.2**	7.1	5.1

**P < 0.01. Pearson's chi-square test of significance.

## Discussion

This study describes the prevalence and clustering patterns of three health behaviours (physical activity, fruit and vegetable consumption, breakfast consumption) in a sample of adolescents from the UK. Almost 54% of adolescents had multiple diet and physical activity risk behaviours, and only 6% achieved the recommendations for all three of the health behaviours. Findings highlight the need for effective strategies promoting multiple healthy lifestyle behaviours.

Higher levels of MVPA were found among boys, compared to girls, and in younger adolescents, compared to older adolescents. Such findings support previous research [27,28] highlighting gender differences and age-related declines in physical activity, using objective measures of physical activity among adolescents. Recent research using accelerometry has also shown that age and gender differences are evident when comparing children as young as six and eleven years of age [29,30]. This suggests that the primary school years may be critical for the development of disparities in physical activity behaviours [31]. Efforts to promote physical activity should begin in these critical years given that active children are more likely to become active adults [32]. Promoting an active lifestyle to girls is challenging, and may require careful consideration of girls' activity preferences with a view to reassessing choices that are made available to them [33].

Consistent with previous research, adolescent girls ate breakfast on fewer days per week compared to boys [13,34,35]. Despite the evidence that adolescents who skip breakfast are more likely to be overweight than those who regularly eat breakfast [5], skipping breakfast may be a chosen method of weight control for girls, and in some individuals may be associated with dieting, or disordered eating [35]. Further research is needed to understand the gender differences in breakfast consumption. Consuming a healthy breakfast on a daily basis should be promoted to young people with an emphasis on educating young girls about the negative health outcomes of breakfast skipping.

Older adolescents ate more fruit and vegetables per day, and were more likely to meet the recommendations for fruit and vegetable consumption, compared to younger adolescents. In contrast, review level evidence has shown a negative association between age and fruit and vegetable consumption [36]. Contrasting findings may reflect a difference in the methodologies employed to assess fruit and vegetable consumption. Several studies have identified overestimation of fruit and vegetable intake when using food frequency questionnaires [37,38]. Although the YAQ is probably the most suitable and well-tested [19,39] tool for assessing dietary intake among adolescents, there are problems assessing dietary intake with self-reported measures. Given that the fruit and vegetable consumption levels reported by participants in this study are higher than those reported by the same aged adolescents in the Health Survey for England [40], our findings may reflect an age-related response bias as well as an over-reporting by adolescents.

The most prevalent of the two-behaviour risk cluster included physical activity and fruit and vegetable consumption and supports previous evidence of such clustering [11,16]. Girls were more likely to have the two-behaviour risk cluster that paired physical activity and breakfast consumption. A possible explanation for such clustering is that adolescents who frequently skip breakfast have lower daily energy intakes, with higher daily energy intake being associated with more time spent being physically active [41]. Girls who skip breakfast as part of a diet or method of weight control, may have less energy to be physically active. Efforts to promote both physical activity and regular breakfast consumption to adolescent girls, in particular, are needed. Consistent with previous research, girls had a higher number of risk factors related to physical activity and dietary behaviours compared to boys [16]. Such findings provide additional evidence for support of gender specific interventions promoting physical activity and dietary behaviours.

Although health promotion programmes frequently target multiple behaviours, little is known about the best approaches to stimulating multiple behaviour change in adolescents [16]. Reviews of multiple behaviour interventions with young people have revealed changes in some but not all behaviours, with significant effects more likely for dietary as opposed to physical activity outcomes [42]. There is little evidence of covariation among diet and physical activity behaviours in adolescents over time [43]. The implication is that multiple health behaviour change interventions may need to address health behaviours individually. Initial research comparing single (physical activity alone) versus multiple behaviour (physical activity and nutrition) interventions found that targeting two health behaviours was associated with less overall behaviour change, compared to targeting one behaviour [44]. However, the intervention targeting both nutrition and physical activity was of low intensity and possibly not sufficient enough to increase fruit and vegetable consumption in the study population [44]. Also, the efficacy of an intervention targeting fruit and vegetables alone was not assessed, so it is not known whether the lack of an effect for fruit and vegetable consumption was due to targeting multiple behaviours concurrently or to the consequence of trying to intervene on a difficult-to-change behaviour [44]. Future research should investigate how best to achieve multiple health behaviour change in adolescent boys and girls.

The present study is innovative in that it examined the potential clustering patterns of breakfast consumption with fruit and vegetable consumption and objectively measured physical activity in a sample of adolescents from the UK. However, we acknowledge that there are a number of limitations that could be usefully addressed in future research. A limitation is the low response rate, reflecting difficulties in fostering compliance when using accelerometers with adolescents [45]. Fruit and vegetable consumption levels reported by participants in this study were higher than the national averages for England, reflecting the difficulties in measuring diet in young people using self-report methods. Such high reported levels of fruit and vegetable consumption may also be reflective of our high SES sample. In the UK there are national differences in fruit and vegetable consumption according to SES, with higher levels of fruit and vegetables being consumed by those from higher SES groups [46]. Staff at participating schools selected a subsample of classes to participate in the study in order to minimise disruption to the school timetable during data collection, this could have led to a selection bias. This study is also limited by its cross-sectional design and the generalisability of the results is limited because participants were predominantly White and of higher SES, thus findings should not be generalised beyond this population.

## Conclusion

Many adolescents fail to meet multiple diet and physical activity recommendations, supporting previous evidence that physical activity and dietary behaviours do not occur in isolation. Differences in dietary and physical activity behaviours between adolescent boys and girls, as well as between older and younger adolescents, should be taken into consideration when assessing the efficacy of strategies promoting multiple health behaviour change.

## Competing interests

The authors declare that they have no competing interests.

## Authors' contributions

NP analyzed the data and conceived and drafted the original manuscript. AJA, SJHB, TG and CE provided critical feedback on drafts. All authors read and approved the final manuscript.
